# CogDrisk, ANU-ADRI, CAIDE, and LIBRA Risk Scores for Estimating Dementia Risk

**DOI:** 10.1001/jamanetworkopen.2023.31460

**Published:** 2023-08-30

**Authors:** Md Hamidul Huque, Scherazad Kootar, Ranmalee Eramudugolla, S. Duke Han, Michelle C. Carlson, Oscar L. Lopez, David A. Bennett, Ruth Peters, Kaarin J. Anstey

**Affiliations:** 1 School of Psychology, University of New South Wales, Kensington, New South Wales, Australia; 2 Neuroscience Research Australia, Randwick, New South Wales, Australia; 3 University of New South Wales Ageing Futures Institute, University of New South Wales, Kensington, New South Wales, Australia; 4 Department of Family Medicine, Keck School of Medicine of the University of Southern California, Los Angeles; 5 Department of Mental Health, Johns Hopkins Bloomberg School of Public Health, and Johns Hopkins Center on Aging and Health, Baltimore, Maryland; 6 Departments of Neurology and Psychiatry, University of Pittsburgh School of Medicine, Pittsburgh, Pennsylvania; 7 Rush Alzheimer’s Disease Center, Rush University Medical Center, Chicago, Illinois; 8 The George Institute of Global Health, University of New South Wales, Kensington, New South Wales, Australia

## Abstract

**Importance:**

While the Australian National University–Alzheimer Disease Risk Index (ANU-ADRI), Cardiovascular Risk Factors, Aging, and Dementia (CAIDE), and Lifestyle for Brain Health (LIBRA) dementia risk tools have been widely used, a large body of new evidence has emerged since their publication. Recently, Cognitive Health and Dementia Risk Index (CogDrisk) and CogDrisk for Alzheimer disease (CogDrisk-AD) risk tools have been developed for the assessment of dementia and AD risk, respectively, using contemporary evidence; comparison of the relative performance of these risk tools is limited.

**Objective:**

To evaluate the performance of CogDrisk, ANU-ADRI, CAIDE, LIBRA, and modified LIBRA (LIBRA with age and sex estimates from ANU-ADRI) in estimating dementia and AD risks (with CogDrisk-AD and ANU-ADRI).

**Design, Setting, and Participants:**

This population-based cohort study obtained data from the Rush Memory and Aging Project (MAP), the Cardiovascular Health Study Cognition Study (CHS-CS), and the Health and Retirement Study–Aging, Demographics and Memory Study (HRS-ADAMS). Participants who were free of dementia at baseline were included. The factors were component variables in the risk tools that included self-reported baseline demographics, medical risk factors, and lifestyle habits. The study was conducted between November 2021 and March 2023, and statistical analysis was performed from January to June 2023.

**Main outcomes and measures:**

Risk scores were calculated based on available factors in each of these cohorts. Area under the receiver operating characteristic curve (AUC) was calculated to measure the performance of each risk score. Multiple imputation was used to assess whether missing data may have affected estimates for dementia risk.

**Results:**

Among the 6107 participants in 3 validation cohorts included for this study, 2184 participants without dementia at baseline were available from MAP (mean [SD] age, 80.0 [7.6] years; 1606 [73.5%] female), 548 participants without dementia at baseline were available from HRS-ADAMS (mean [SD] age, 79.5 [6.3] years; 288 [52.5%] female), and 3375 participants without dementia at baseline were available from CHS-CS (mean [SD] age, 74.8 [4.9] years; 1994 [59.1%] female). In all 3 cohorts, a similar AUC for dementia was obtained using CogDrisk, ANU-ADRI, and modified LIBRA (MAP cohort: CogDrisk AUC, 0.65 [95% CI, 0.61-0.69]; ANU-ADRI AUC, 0.65 [95% CI, 0.61-0.69]; modified LIBRA AUC, 0.65 [95% CI, 0.61-0.69]; HRS-ADAMS cohort: CogDrisk AUC, 0.75 [95% CI, 0.71-0.79]; ANU-ADRI AUC, 0.74 [95% CI, 0.70-0.78]; modified LIBRA AUC, 0.75 [95% CI, 0.71-0.79]; CHS-CS cohort: CogDrisk AUC, 0.70 [95% CI, 0.67-0.72]; ANU-ADRI AUC, 0.69 [95% CI, 0.66-0.72]; modified LIBRA AUC, 0.70 [95% CI, 0.68-0.73]). The CAIDE and LIBRA also provided similar but lower AUCs than the 3 aforementioned tools (eg, MAP cohort: CAIDE AUC, 0.50 [95% CI, 0.46-0.54]; LIBRA AUC, 0.53 [95% CI, 0.48-0.57]). The performance of CogDrisk-AD and ANU-ADRI in estimating AD risks was also similar.

**Conclusions and relevance:**

CogDrisk and CogDrisk-AD performed similarly to ANU-ADRI in estimating dementia and AD risks. These results suggest that CogDrisk and CogDrisk-AD, with a greater range of modifiable risk factors compared with other risk tools in this study, may be more informative for risk reduction.

## Key Points

**Question:**

How do newly available assessment tools, Cognitive Health and Dementia Risk Index (CogDrisk) and CogDrisk for Alzheimer disease (CogDrisk-AD), perform compared with existing tools for estimating dementia and Alzheimer disease risks?

**Findings:**

In this cohort study of 6107 participants from 3 validation cohorts, the performance of CogDrisk and CogDrisk-AD was similar to the Australian National University–Alzheimer Disease Risk Index in estimating dementia and Alzheimer disease risks.

**Meaning:**

As CogDrisk and CogDrisk-AD are based on more up-to-date evidence and include more modifiable risk factors relative to other risk scores in this study, they may have more potential to inform risk modification and/or risk intervention in the community.

## Teaser

This cohort study evaluates the performance of the Cognitive Health and Dementia Risk Index (CogDrisk), the Australian National University–Alzheimer Disease Risk Index (ANU-ADRI), the Cardiovascular Risk Factors, Aging, and Dementia (CAIDE), the Lifestyle for Brain Health (LIBRA), and modified LIBRA (LIBRA with age and sex estimates from ANU-ADRI) in estimating dementia risks; and CogDrisk for Alzheimer disease (CogDrisk-AD) and ANU-ADRI in estimating Alzheimer disease risks.

## Introduction

Globally, dementia is a leading cause of disability and dependency among older people, with more than 55 million people living with dementia worldwide.^1^ To date, there is no effective and widely available treatment for dementia, and this has resulted in strategies that have aimed to improve and to raise awareness of dementia risk factors among patients and health practitioners. Several dementia and Alzheimer disease (AD) risk assessment tools have been developed for use in community settings without optimization for use in day-to-day clinical practice, or in routine clinical consultation.^2,3,4,5^ Of the available risk tools, the Cognitive Health and Dementia Risk Index (CogDrisk), CogDrisk for AD (CogDrisk-AD),^2^ and the Australian National University–Alzheimer Disease Risk Index (ANU-ADRI),^3^ were developed using data collated from an evidence synthesis approach. Of 2 other dementia risk tools that are both well established in the literature, the Cardiovascular Risk Factors, Aging, and Dementia (CAIDE)^5^ was developed from a single cohort, and the Lifestyle for Brain Health (LIBRA)^4^ was developed based on the results of a systematic literature review and Delphi expert consensus study. The CogDrisk tool was developed to estimate dementia risks, and the CogDrisk-AD tool estimates AD risks. The ANU-ADRI, although originally developed to estimate AD risks, also exhibits good estimates for dementia risks.^6^ All of the aforementioned tools have been validated in various cohorts and exhibit good ability to estimate risks for dementia.^6,7,8,9,10^ Perhaps because these tools differ in their construction and the methodology used in their development, direct comparisons have thus far been limited. While a few studies have compared risk tools in a single cohort,^11,12,13^ to our knowledge, there has been no thorough comparison of the tools across multiple independent cohorts. In order to facilitate the implementation of risk scores in primary care and public health practice, we need to understand what each tool requires to allow calculation of a full score, and how well each tool performs in estimating dementia or AD risks in a range of populations. In this study, we aimed to perform a direct comparison of the risk tools by applying them to the same data sets simultaneously in order to provide necessary information for comparing performance and guiding future implementation.

## Methods

In this cohort study, we considered 3 well-known, population-based cohorts with dementia for the validation of 4 risk tools for dementia and 2 risk tools for AD. The study was conducted between November 2021 and March 2023 and followed the Strengthening the Reporting of Observational Studies in Epidemiology (STROBE) reporting guideline.^14^ Ethical approval for this study was obtained from the University of New South Wales Human Research Ethics Committee. Each of the individual cohorts were approved by their respective institutional review boards. All participants provided informed consent to be included in the original studies. As we are using secondary data analysis, informed consent was not necessary for our study.

### Population-Based Cohorts for Validation of Risk Tools

#### The Rush Memory and Aging Project

The Rush Memory and Aging Project (MAP) included 2184 participants aged 60 years and older from the greater Chicago area. The study has a rolling recruitment from 1997 to present. Up to 22 years of follow-up data are available. Dementia was diagnosed using a computer algorithm.^15^ The study was approved by the Rush University Medical Center institutional review board. All participants signed informed and repository consents.

#### The Health and Retirement Study—Aging, Demographics, and Memory Study (HRS-ADAMS)

The Health and Retirement Study—Aging, Demographics, and Memory Study (HRS-ADAMS) is a supplementary study in the HRS that conducted in-person clinical assessments to gather information about cognitive status.^16^ The clinical diagnosis of dementia was based on the *Diagnostic and Statistical Manual of Mental Disorders* (Third Edition Revised) and *Diagnostic and Statistical Manual of Mental Disorders* (Fourth Edition). The study consisted of 856 community-based individuals aged at least 70 years (including prevalent dementia cases) throughout the US who were assessed in 2001 (baseline) and followed up through to 2008. In our current study, 308 participants with prevalent dementia were excluded.

#### The Cardiovascular Health Study Cognition Study

The Cardiovascular Health Study Cognition Study (CHS-CS) was an ancillary study of the main Cardiovascular Health Study. The CHS-CS was initiated in 1991 to 1994 using amplified magnetic resonance imaging of the brain and was followed up until 1999 to 2000 with 3602 community-based participants from 4 states in the US.^17^ Participants were evaluated for dementia by detailed neurological and neuropsychological examinations. Clinical diagnosis of dementia was ascertained by a review committee of neurologists and psychiatrists.^17^ In our current study, 227 participants with dementia at baseline were excluded.

### Risk Tools Included for Analysis

#### CogDrisk and CogDrisk-AD

The CogDrisk and CogDrisk-AD risk tools were developed for the assessment of dementia risk and AD risk, respectively, for adults aged 65 years and over.^2^ CogDrisk was developed using 17 established dementia risk factors identified through systematic review and meta-analysis (eTable 1 in Supplement 1). Following systematic review and meta-analysis, risk ratios were obtained for each of the risk factors, which were then converted to points and summed to develop a risk score.^2,3^ The same method was used to develop CogDrisk-AD with 16 risk factors associated with AD.^2^

#### ANU-ADRI

The ANU-ADRI was also developed to estimate AD risk in public health settings for adults aged 65 years and above.^3^ The ANU-ADRI assesses the presence of 11 risk factors for AD and 4 factors associated with reduced risk for AD. Similar to the CogDrisk-AD, the ANU-ADRI identifies factors associated with increased or decreased risk for AD through evidence synthesis, and risk scores were calculated based on points derived from the risk ratios.

#### LIBRA

The LIBRA dementia risk tool was developed from a review of the literature and a Delphi consensus study.^4^ The tool includes only modifiable risk factors at midlife. The algorithm for LIBRA was developed from the risk scores obtained from the relative risk of all factors identified in a systematic review. The LIBRA score was later validated using the Rotterdam Study, and the authors suggested a modified LIBRA risk tool that includes weights for age, sex, and education from ANU-ADRI.^13^

#### CAIDE

The CAIDE score was developed in a Finnish population-based cohort, aged 39 to 64 years at baseline, who were reexamined for the signs of dementia 20 years later. It uses vascular risk factors at midlife (see eTable 1 in Supplement 1 for risk factor information) along with age, gender, and apolipoprotein E ε4 allele (*APOE* ε4) status. The algorithm for CAIDE risk score was developed using the logistic regression coefficients for the risk factors.

### Statistical Analysis

For each of the cohorts under assessment, risk factors were either taken from baseline or the follow-up closest to baseline (where baseline assessment was not available) under the assumption that the specific characteristics were constant over time. The points associated with all the available risk factors in each cohort were added to calculate the risk score. The accuracy of the risk scores for identifying participants at risk of dementia and AD was quantified by calculating the area under the receiver operating characteristic curves (AUCs) and associated 95% CIs.^18^ To assess the association of modifiable risk factors with dementia, the AUCs of the risk scores were further evaluated without points for age, sex, and education. For a given cutoff (quantile ranks) of the risk scores, we also compared relative odds ratios, sensitivity, and specificity across all cohorts.

To assess whether missing data may have affected estimates for dementia risk, we ran 2 different sensitivity analyses: (1) reduced variable analysis: reduced model by removing factors associated with increased or decreased risk with a large number of missing observations (ranging from 24% to 61%), which improved the sample sizes; and (2) multiple imputation. To carry out multiple imputation, we excluded participants who had missing education status or who had missing data on more than 5 covariates (exclusions: 8 participants in MAP, 1 in HRS-ADAMS, and 5 in CHS-CS). We considered multivariate normal imputation with 20 imputed data sets.^19^ In the multiple imputation models, appropriate covariates were included in the imputation model to ensure compatibility between the imputation and analysis model. All analyses were conducted using Stata statistical software version 16.0 (StataCorp) from January to June 2023.

## Results

### Description of the 3 Validation Cohorts

Among the 3 cohorts included for this study, 2184 participants without dementia at baseline were available from MAP (mean [SD] baseline age, 80.0 [7.6] years), 548 participants without dementia at baseline were available from HRS-ADAMS (mean [SD] age, 79.5 [6.3] years), and 3375 participants without dementia at baseline were available from CHS-CS (mean [SD] age, 74.8 [4.9] years) (eTable 2 in Supplement 1). A majority of the participants in all 3 cohorts were female (1606 [73.5%] in MAP, 1994 [59.1%] in CHS-CS, and 288 [52.5%] in HRS-ADAMS). A majority of the participants in the MAP (2027 [92.8%]) and CHS-CS (2560 [75.9%]) cohorts had a tertiary education. MAP was the only cohort that had a small number of participants (80 [3.7%]) from midlife (aged <65 years); midlife risk factors, such as obesity and hypertension, were evaluated only for these participants in the calculation of risk scores. Social engagement was also available only for the MAP cohort, whereas kidney dysfunction was only available for the CHS-CS cohort. None of the cohorts had information on midlife high cholesterol or pesticide exposure. Overall, the 3 cohorts differed in terms of risk factors assessed and their prevalence at baseline. The number of incident dementia cases available in these data sets were 106 (19.3%) for HRS-ADAMS, 589 (27.0%) for MAP, and 480 (14.2%) for CHS-CS. The number of incident AD cases available for these data sets were 571 (26.1%) for MAP, 77 (14.1%) for HRS-ADAMS, and 396 (11.7%) for CHS-CS. Median (IQR) follow-up times were 5.0 (1.0-2.0) years for MAP, 5.0 (2.0-6.0) years for HRS-ADAMS, and 6.0 (0.2-7.7) years for CHS-CS.

### Performance of the Dementia Risk Tools for Estimating Dementia Risks

We report the results of the analyses comparing the relative accuracy of CogDrisk, ANU-ADRI, CAIDE, LIBRA, and modified LIBRA for estimating dementia risks in the MAP cohort (Table 1), HRS-ADAMS cohort (Table 2), and CHS-CS cohort (Table 3). Overall, the performance of CogDrisk, ANU-ADRI, and modified LIBRA were very similar in estimating dementia risks when applied to the available data in all 3 cohorts (MAP cohort: CogDrisk AUC, 0.65 [95% CI, 0.61-0.69]; ANU-ADRI AUC, 0.65 [95% CI, 0.61-0.69]; modified LIBRA AUC, 0.65 [95% CI, 0.61-0.69]; HRS-ADAMS cohort: CogDrisk AUC, 0.75 [95% CI, 0.71-0.79]; ANU-ADRI AUC, 0.74 [95% CI, 0.70-0.78]; modified LIBRA AUC, 0.75 [95% CI, 0.71-0.79]; CHS-CS cohort: CogDrisk AUC, 0.70 [95% CI, 0.67-0.72]; ANU-ADRI AUC, 0.69 [95% CI, 0.66-0.72]; modified LIBRA AUC, 0.70 [95% CI, 0.68-0.73]). The AUCs of CAIDE and LIBRA were comparatively lower than the aforementioned 3 risk scores in all 3 cohorts (MAP cohort: CAIDE AUC, 0.50 [95% CI, 0.46-0.54]; LIBRA AUC, 0.53 [95% CI, 0.48-0.57]; HRS-ADAMS cohort: CAIDE AUC, 0.56 [95% CI, 0.49-0.63]; LIBRA AUC, 0.52 [95% CI, 0.45-0.59]; CHS-CS cohort: CAIDE AUC, 0.57 [95% CI, 0.52-0.61]; LIBRA AUC, 0.51 [95% CI, 0.48-0.54]) (Table 1, Table 2, and Table 3).

**Table 1.** Comparison of the Performance of 5 Risk Scores for Estimating Dementia Risks in MAP Cohort

All variables model^a^	Risk score, AUC (95% CI)				
	CogDrisk	ANU-ADRI	CAIDE	LIBRA	Modified LIBRA
Available data analysis (N = 843 [209 male, 634 female])					
Male	0.66 (0.58-0.73)	0.61 (0.53-0.69)	0.56 (0.49-0.63)	0.58 (0.49-0.66)	0.63 (0.56-0.71)
Female	0.65 (0.61-0.70)	0.66 (0.62-0.71)	0.48 (0.44-0.52)	0.51 (0.46-0.56)	0.66 (0.61-0.70)
Overall	0.65 (0.61-0.69)	0.65 (0.61-0.69)	0.50 (0.46-0.54)	0.53 (0.48-0.57)	0.65 (0.61-0.69)
Multiple imputed analysis (N = 2172 [574 male, 1598 female])					
Male	0.66 (0.62-0.71)	0.64 (0.59-0.69)	0.53 (0.49-0.58)	0.56 (0.51-0.61)	0.66 (0.61-0.70)
Female	0.64 (0.61-0.67)	0.65 (0.62-0.68)	0.51 (0.48-0.54)	0.49 (0.46-0.52)	0.65 (0.63-0.68)
Overall	0.65 (0.63-0.68)	0.64 (0.62-0.67)	0.51 (0.48-0.54)	0.53 (0.50-0.55)	0.65 (0.63-0.68)
**Reduced variables model** ^b^					
Available data analysis (N = 1472 [367 male, 1105 female])					
Male	0.68 (0.62-0.73)	0.66 (0.60-0.71)	0.54 (0.49-0.60)	0.54 (0.48-0.61)	0.66 (0.60-0.72)
Female	0.65 (0.62-0.69)	0.67 (0.63-0.70)	0.48 (0.45-0.52)	0.52 (0.48-0.55)	0.66 (0.62-0.69)
Overall	0.66 (0.63-0.69)	0.66 (0.63-0.69)	0.50 (0.47-0.53)	0.52 (0.49-0.55)	0.66 (0.63-0.69)
Multiple imputed analysis (N = 2172 [574 male, 1598 female])					
Male	0.67 (0.62-0.71)	0.65 (0.60-0.70)	0.53 (0.49-0.58)	0.56 (0.51-0.61)	0.66 (0.61-0.70)
Female	0.64 (0.61-0.67)	0.65 (0.62-0.68)	0.51 (0.48-0.54)	0.49 (0.46-0.52)	0.65 (0.63-0.68)
Overall	0.65 (0.63-0.68)	0.64 (0.62-0.67)	0.51 (0.48-0.54)	0.53 (0.50-0.55)	0.65 (0.63-0.68)

Abbreviations: ANU-ADRI, Australian National University–Alzheimer Disease Risk Index; AUC, area under the receiver operating characteristic curve; CAIDE, Cardiovascular Risk Factors, Aging, and Dementia; CogDrisk, Cognitive Health and Dementia Risk Index; LIBRA, Lifestyle for Brain Health; MAP, Rush Memory and Aging Project.

^a^ In the all variables model, CogDrisk included 14 factors associated with increased or reduced risk: age, gender, education, obesity, diabetes, depression, traumatic brain injury, smoking, loneliness, physical activity, cognitive activity, fish intake, stroke, and hypertension; ANU-ADRI included 13 factors: age, gender, education, obesity, diabetes, depression, traumatic brain injury, smoking, social network, physical activity, cognitive activity, fish intake, and alcohol intake; CAIDE included 7 factors: age, gender, education, obesity, traumatic brain injury, physical activity, and hypertension; LIBRA included 9 factors: obesity, diabetes, depression, smoking, physical activity, cognitive activity, alcohol intake, hypertension, and coronary heart disease; modified LIBRA included 12 factors: age, gender, education, obesity, diabetes, depression, smoking, physical activity, cognitive activity, alcohol intake, hypertension, and coronary heart disease.

^b^ In the reduced variables model, CogDrisk included 13 factors associated with increased or reduced risk: age, gender, education, obesity, diabetes, depression, traumatic brain injury, smoking, loneliness, physical activity, cognitive activity, stroke, and hypertension; ANU-ADRI included 12 factors: age, gender, education, obesity, diabetes, depression, traumatic brain injury, smoking, social network, physical activity, cognitive activity, and alcohol intake; CAIDE included 7 factors: age, gender, education, obesity, traumatic brain injury, physical activity, and hypertension; LIBRA included 9 factors: obesity, diabetes, depression, smoking, physical activity, cognitive activity, alcohol intake, hypertension, and coronary heart disease; modified LIBRA included 12 factors: age, gender, education, obesity, diabetes, depression, smoking, physical activity, cognitive activity, alcohol intake, hypertension, and coronary heart disease. Obesity and hypertension were only calculated for observations of those aged less than 65 years.

**Table 2.** Comparison of the Performance of 5 Risk Scores for Estimating Dementia Risk in HRS-ADAMS Cohort

All variables model^a^	Risk score, AUC (95% CI)				
	CogDrisk	ANU-ADRI	CAIDE	LIBRA	Modified LIBRA
Available data analysis (N = 421 [200 male, 221 female])					
Male	0.71 (0.60-0.82)	0.68 (0.57-0.80)	0.62 (0.51-0.73)	0.50 (0.39-0.61)	0.69 (0.58-0.81)
Female	0.64 (0.55-0.73)	0.63 (0.52-0.72)	0.58 (0.49-0.67)	0.57 (0.48-0.66)	0.64 (0.55-0.73)
Overall	0.65 (0.58-0.72)	0.66 (0.59-0.73)	0.56 (0.49-0.63)	0.52 (0.45-0.59)	0.67 (0.61-0.74)
Multiple imputed analysis (N = 547 [260 male, 287 female])					
Male	0.67 (0.58-0.76)	0.67 (0.58-0.75)	0.59 (0.50-0.68)	0.56 (0.47-0.66)	0.67 (0.58-0.76)
Female	0.62 (0.54-0.70)	0.60 (0.52-0.68)	0.57 (0.49-0.64)	0.52 (0.44-0.60)	0.62 (0.54-0.70)
Overall	0.64 (0.58-0.69)	0.63 (0.58-0.69)	0.55 (0.49-0.61)	0.53 (0.47-0.58)	0.65 (0.59-0.71)
**Reduced variables model^b^**					
Available data analysis (N = 432 [205 male, 227 female])					
Male	0.67 (0.56-0.79)	0.68 (0.57-0.79)	0.58 (0.47-0.69)	0.44 (0.34-0.55)	0.69 (0.57-0.80)
Female	0.64 (0.55-0.73)	0.64 (0.55-0.73)	0.59 (0.51-0.67)	0.60 (0.51-0.69)	0.65 (0.56-0.74)
Overall	0.64 (0.57-0.71)	0.66 (0.59-0.73)	0.55 (0.48-0.63)	0.53 (0.46-0.60)	0.67 (0.61-0.74)
Multiple imputed analysis (N = 547 [260 male, 287 female])					
Male	0.65 (0.56-0.74)	0.66 (0.58-0.75)	0.57 (0.47-0.66)	0.56 (0.47-0.66)	0.67 (0.58-0.76)
Female	0.61 (0.53-0.69)	0.61 (0.53-0.69)	0.58 (0.50-0.65)	0.53 (0.45-0.61)	0.63 (0.55-0.70)
Overall	0.62 (0.56-0.68)	0.64 (0.58-0.70)	0.56 (0.49-0.62)	0.51 (0.45-0.57)	0.65 (0.59-0.71)

Abbreviations: ANU-ADRI, Australian National University–Alzheimer Disease Risk Index; AUC, area under the receiver operating characteristic curve; CAIDE, Cardiovascular Risk Factors, Aging, and Dementia; CogDrisk, Cognitive Health and Dementia Risk Index; HRS-ADAMS, Health and Retirement Study–Aging, Demographics and Memory Study; LIBRA, Lifestyle for Brain Health.

^a^ In the all variables model, CogDrisk included 10 factors associated with increased or reduced risk: age, gender, education, diabetes, depression, traumatic brain injury, smoking, cognitive activity, insomnia, and stroke; ANU-ADRI included 9 factors: age, gender, education, diabetes, depression, traumatic brain injury, smoking, cognitive activity, and alcohol intake; CAIDE included 5 factors: age, gender, education, traumatic brain injury, and hypertension; LIBRA included 7 factors: diabetes, depression, smoking, cognitive activity, alcohol intake, hypertension, and coronary heart disease; modified LIBRA included 10 factors: age, gender, education, diabetes, depression, smoking, cognitive activity, alcohol intake, hypertension, and coronary heart disease.

^b^ In the reduced variables model, CogDrisk included 8 factors associated with increased or reduced risk: age, gender, education, diabetes, depression, traumatic brain injury, smoking, and cognitive activity; ANU-ADRI included 8 factors: age, gender, education, diabetes, depression, traumatic brain injury, smoking, and cognitive activity; CAIDE included 4 factors: age, gender, education, and traumatic brain injury; LIBRA included 5 factors: diabetes, depression, smoking, physical activity, and cognitive activity; modified LIBRA included 8 factors: age, gender, education, diabetes, depression, smoking, and cognitive activity.

**Table 3.** Comparison of the Performance of 5 Risk Scores for Estimating Dementia Risk in CHS-CS Cohort

All variables model^a^	Risk score, AUC (95% CI)					
	CogDrisk	ANU-ADRI		CAIDE	LIBRA	Modified LIBRA
Available data analysis (N = 3097 [1263 male, 1834 female])						
Male	0.70 (0.66-0.74)		0.68 (0.64-073)	0.62 (0.54-0.69)	0.52 (0.47-0.55)	0.70 (0.65-0.74)
Female	0.70 (0.66-0.73)		0.69 (0.66-0.73)	0.59 (0.53-0.65)	0.51 (0.47-0.57)	0.71 (0.68-0.75)
Overall	0.70 (0.67-0.72)		0.69 (0.66-0.72)	0.57 (0.52-0.61)	0.51 (0.48-0.54)	0.70 (0.68-0.73)
Multiple imputed analysis (N = 3370 [1381 male, 1989 female])						
Male	0.70 (0.66-0.74)		0.68 (0.64-0.72)	0.57 (0.53-0.61)	0.56 (0.52-0.61)	0.69 (0.65-0.73)
Female	0.71 (0.67-0.74)		0.70 (0.67-0.73)	0.56 (0.52-0.59)	0.52 (0.49-0.56)	0.71 (0.68-0.75)
Overall	0.70 (0.68-0.73)		0.69 (0.67-0.72)	0.56 (0.53-0.58)	0.54 (0.51-0.57)	0.70 (0.68-0.73)
**Reduced variables model^b^**						
Available data analysis (N = 3273 [1323 male, 1950 female])						
Male	0.70 (0.66-0.74)		0.68 (0.63-0.72)	0.57 (0.53-0.61)	0.51 (0.46-0.56)	0.69 (0.65-0.73)
Female	0.70 (0.66-0.73)		0.69 (0.66-73)	0.56 (0.52-0.59)	0.51 (0.47-0.54)	0.71 (0.68-0.75)
Overall	0.70 (0.67-0.72)		0.69 (0.66-0.71)	0.55 (0.52-0.58)	0.51 (0.48-0.54)	0.70 (0.68-0.73)
Multiple imputed analysis (N = 3370 [1381 male, 1989 female])						
Male	0.70 (0.66-0.74)		0.68 (0.64-0.72)	0.57 (0.53-0.61)	0.56 (0.51-0.60)	0.69 (0.64-0.73)
Female	0.71 (0.67-0.74)		0.70 (0.66-0.73)	0.56 (0.52-0.59)	0.53 (0.49-0.56)	0.71 (0.68-0.75)
Overall	0.70 (0.68-0.73)		0.69 (0.66-0.72)	0.56 (0.53-0.58)	0.54 (0.51-0.57)	0.70 (0.68-0.73)

Abbreviations: ANU-ADRI, Australian National University–Alzheimer Disease Risk Index; AUC, area under the receiver operating characteristic curve; CAIDE, Cardiovascular Risk Factors, Aging, and Dementia; CHS-CS, Cardiovascular Health Study Cognition Study; CogDrisk, Cognitive Health and Dementia Risk Index; LIBRA, Lifestyle for Brain Health.

^a^ In the all variables model, CogDrisk included 12 factors associated with increased or reduced risk: age, gender, education, diabetes, depression, smoking, physical activity, loneliness, insomnia, stroke, atrial fibrillation, and fish intake; ANU-ADRI included 9 factors: age, gender, education, diabetes, depression, smoking, alcohol intake, physical activity, and fish intake; CAIDE included 4 factors: age, gender, education, physical activity; LIBRA included 7 factors: diabetes, depression, smoking, alcohol intake, physical activity, coronary heart disease, and kidney dysfunction; modified LIBRA included 10 factors: age, gender, education, diabetes, depression, smoking, alcohol intake, physical activity, coronary heart disease, and kidney dysfunction.

^b^ In the reduced variables model, CogDrisk included 11 factors associated with increased or reduced risk: age, gender, education, depression, smoking, physical activity, loneliness, insomnia, stroke, atrial fibrillation, and fish intake; ANU-ADRI included 8 factors: age, gender, education, depression, smoking, alcohol intake, physical activity, and fish intake; CAIDE included 4 factors: age, gender, education, and physical activity; LIBRA included 5 factors: depression, smoking, alcohol intake, physical activity, and coronary heart disease; modified LIBRA included 8 factors: age, gender, education, depression, smoking, alcohol intake, physical activity, and coronary heart disease.

Dementia risk estimates were better among female participants in the MAP study using CogDrisk, ANU-ADRI, and modified LIBRA, whereas CAIDE and LIBRA had better risk estimates among male participants (Table 1). In the HRS-ADAMS, better AUCs were obtained for male participants except for LIBRA, which estimated dementia risks in female participants better than in male participants (Table 2). In CHS-CS, similar AUCs were obtained for both male and female participants (Table 3). For multiple imputed data, the results were very similar to that of available data analysis across all 3 cohorts. The reduced variable analysis also resulted in similar AUCs to that in the available data analysis.

### Performance of CogDrisk, ANU-ADRI, CAIDE, and LIBRA Risk Scores Without Age, Sex, and Education for Estimating Dementia Risks 

eTable 3 in Supplement 1 reports the results of the analyses comparing the AUC and 95% CI of CogDrisk, ANU-ADRI, CAIDE, and LIBRA for estimating dementia risks on all 3 cohorts where risk scores were calculated without age, sex, and education. The 4 risk scores resulted in very similar AUCs for dementia when considered without age, sex, and education.

### Comparison of Sensitivity, Specificity, and Cutoff Points of CogDrisk, ANU-ADRI, CAIDE, LIBRA, and Modified LIBRA Risk Scores

The final risk scores for all 5 risk tools varies across all 3 cohorts. Yet, we observed positive correlation between scores obtained by CogDrisk, ANU-ADRI, and modified LIBRA (eg, correlation coefficient between CogDrisk and ANU-ADRI is 0.89 in MAP cohort, 0.94 in HRS-ADAMS, and 0.83 in CHS-CS; correlation coefficient between CogDrisk and modified LIBRA is 0.86 in MAP, 0.85 in HRS-ADAMS, and 0.86 in CHS-CS; and correlation coefficient between ANU-ADRI and modified LIBRA is 0.92 in MAP, 0.94 in HRS-ADAMS, and 0.96 in CHS-CS.) (eTable 4 in Supplement 1). These 3 risk scores remained correlated when calculated without the weights for age, sex, and education (eTable 5 in Supplement 1).

Table 4 reports odds ratios (ORs), sensitivity, and specificity associated with 95% CI of various cutoff values for each of the 5 risk scores in MAP, HRS-ADAMS, and CHS-CS. The cutoff values were determined based on quantile ranks of risk scores. Overall, the OR increased for higher quantile cutoffs for CogDrisk, ANU-ADRI, and modified LIBRA in HRS-ADAMS and CHS-CS. Although the actual risk scores for cutoffs varied across cohorts, a median cutoff (score ≥50%) provided approximately 70% sensitivity and using a cutoff of greater than 33% provided approximately 80% sensitivity for CogDrisk, ANU-ADRI, and modified LIBRA in all 3 cohorts.

**Table 4.** Comparison of Sensitivity, Specificity for a Given Cutoff of CogDrisk, ANU-ADRI, CAIDE, and LIBRA Risk Scores in All 3 Cohorts

Risk score quantile cutoff	MAP				HRS-ADAMS				CHS-CS			
	Cutoff score	OR (95% CI)	Sensitivity, % (95% CI)	Specificity, % (95% CI)	Cutoff score	OR (95% CI)	Sensitivity, % (95% CI)	Specificity, % (95% CI)	Cutoff score	OR (95% CI)	Sensitivity, % (95% CI)	Specificity, % (95% CI)
CogDrisk cutoff, %												
≥16.6	3.7	3.99 (2.03 to 7.82)	95.0 (91.6 to 97.3)	22.4 (19.1 to 26.0)	11	1.09 (0.40 to 3.00)	89.3 (80.1 to 95.3)	18.8 (14.8 to 23.3)	1.9	0.77 (0.44 to 1.35)	92.2 (89.0 to 94.7)	18.1 (16.6 to 19.7)
≥33.3	7.5	3.93 (1.99 to 7.75)	78.8 (73.3 to 83.6)	40.6 (36.6 to 44.7)	14	1.35 (0.52 to 3.52)	77.3 (66.2 to 86.2)	38.2 (33.0 to 43.5)	7.1	1.56 (0.96 to 2.54)	86.1 (82.1 to 89.4)	36.8 (34.9 to 38.7)
≥50	10.5	6.02 (3.10 to 11.71)	63.7 (57.5 to 69.6)	57.7 (53.6 to 61.8)	17	0.86 (0.28 to 2.61)	61.3 (49.4 to 72.4)	59.0 (53.6 to 64.2)	9.5	2.33 (1.47 to 3.70)	74.5 (69.8 to 78.9)	54.0 (52.0 to 56.0)
≥66.7	12.7	5.26 (2.69 to 10.25)	43.6 (37.5 to 49.9)	72.6 (68.8 to 76.2)	19	3.25 (1.30 to 8.12)	53.3 (41.4 to 64.9)	75.4 (70.5 to 79.9)	12.1	3.15 (2.01 to 4.92)	57.6 (52.4 to 62.7)	70.9 (69.0 to 72.7)
≥83.3	16.5	9.63 (4.96 to 18.70)	25.1 (19.9 to 30.8)	88.4 (85.5 to 90.8)	23	4.47 (1.82 to 11.00)	29.3 (19.4 to 41.0)	88.4 (84.6 to 91.6)	15.7	6.31 (4.12 to 9.65)	36.5 (31.6 to 41.6)	86.6 (85.1 to 87.9)
ANU-ADRI cutoff, %												
≥16.6	8	2.17 (1.17 to 4.02)	93.1 (89.2 to 95.8)	22.1 (18.8 to 25.7)	17	1.34 (0.51 to 3.52)	89.3 (80.1 to 95.3)	21.1 (16.9 to 25.8)	7	0.87 (0.51 to 1.48)	91.7 (88.4 to 94.3)	18.3 (16.8 to 19.9)
≥33.3	16	3.50 (1.88 to 6.51)	79.2 (73.7 to 83.9)	42.5 (38.4 to 46.6)	23	1.14 (0.37 to 3.49)	74.7 (63.3 to 84.0)	42.8 (37.5 to 48.2)	11	1.58 (1.00 to 2.50)	84.5 (80.4 to 88.0)	36.7 (34.8 to 38.6)
≥50	21	4.28 (2.36 to 7.77)	63.7 (57.5 to 69.6)	56.5 (52.4 to 60.6)	26	2.24 (0.87 to 5.78)	66.7 (54.8 to 77.1)	56.7 (51.2 to 61.9)	15	1.66 (1.02 to 2.71)	69.2 (64.2 to 73.8)	58.0 (56.0 to 59.9)
≥66.7	26	3.12 (1.70 to 5.73)	42.5 (36.4 to 48.7)	72.3 (68.4 to 75.9)	31	2.32 (0.91 to 5.93)	49.3 (37.6 to 61.1)	72.0 (66.9 to 76.6)	18	3.44 (2.24 to 5.28)	58.4 (53.3 to 63.5)	72.2 (70.4 to 74.0)
≥83.3	32	7.75 (4.24 to 14.18)	25.5 (20.3 to 31.2)	89.6 (86.8 to 91.9)	37	5.00 (2.05 to 2.16)	30.7 (20.5 to 42.4)	87.9 (83.9 to 91.1)	24	6.23 (4.10 to 9.48)	33.8 (29.0 to 38.8)	88.0 (86.7 to 89.3)
CAIDE cutoff, %												
≥16.6	NA	NA	NA	NA	5	0.41 (0.15 to 1.11)	80.0 (69.2 to 88.4)	19.9 (15.9 to 24.5)	5	1.07 (0.82 to 1.39)	73.2 (68.4 to 77.6)	31.2 (29.4 to 33.1)
≥33.3	5	0.87 (0.63 to 1.20)	51.0 (44.7 to 57.2)	48.0 (43.8 to 52.1)	6	1.15 (0.54 to 2.46)	72.0 (60.4 to 81.8)	39.6 (34.4 to 45.0)	NA	NA	NA	NA
≥50.0	NA	NA	NA	NA	7	0.98 (0.47 to 2.07)	48.0 (36.3 to 59.8)	60.4 (55.0 to 65.6)	NA	NA	NA	NA
≥66.7	NA	NA	NA	NA	NA	NA	NA	NA	6	1.26 (0.88 to 1.82)	30.3 (25.7 to 35.2)	78.0 (76.3 to 79.6)
≥83.3	6	1.18 (0.78 to 1.78)	18.1 (13.6 to 23.4)	84.9 (81.8 to 87.7)	8	1.63 (0.74 to 3.58)	22.7 (13.8 to 33.8)	86.1 (82.0 to 89.6)	7	2.04 (1.44 to 2.88)	16.9 (13.2 to 21.1)	90.3 (89.1 to 91.5)
LIBRA cutoff, %												
≥16.6	−0.9	1.92 (1.22 to 3.00)	86.5 (81.7 to 90.4)	20.2 (17.0 to 23.7)	0.5	0.97 (0.41 to 2.32)	84.0 (73.7 to 91.4)	18.8 (14.8 to 23.3)	0.1	1.21 (0.84 to 1.75)	78.3 (73.7 to 82.4)	23.4 (21.7 to 25.1)
≥33.3	NA	NA	NA	NA	1.5	1.35 (0.55 to 3.34)	68.0 (56.2 to 78.3)	38.2 (33.0 to 43.5)	1.0	1.31 (0.91 to 1.89)	63.0 (57.9 to 67.9)	36.9 (35.0 to 38.8)
≥50.0	0.1	1.42 (0.87 to 2.33)	47.9 (41.7 to 54.1)	50.3 (46.2 to 54.5)	1.6	1.68 (0.75 to 3.79)	53.3 (41.4 to 64.9)	50.9 (45.5 to 56.3)	1.5	0.69 (0.47 to 1.01)	47.5 (42.3 to 52.7)	49.6 (47.6 to 51.6)
≥66.7	1.2	1.33 (0.77 to 2.28)	27.0 (21.7 to 32.9)	72.3 (68.4 to 75.9)	2.5	0.98 (0.43 to 2.24)	29.3 (19.4 to 41.0)	67.6 (62.4 to 72.5)	2.1	1.31 (0.78 to 1.60)	35.4 (30.5 to 40.5)	68.5 (66.7 to 70.4)
≥83.3	2.2	1.62 (0.93 to 2.81)	13.5 (9.6 to 18.3)	87.5 (84.5 to 90.1)	3.3	1.31 (0.47 to 3.66)	9.3 (3.8 to 18.3)	91.6 (88.2 to 94.3)	2.7	1.11 (0.78 to 1.59)	16.4 (12.7 to 20.5)	84.2 (82.7 to 85.6)
Modified LIBRA cutoff, %												
≥16.6	14.1	3.56 (1.89 to 6.69)	94.2 (90.6 to 96.7)	22.1 (18.8 to 25.7)	18.1	2.51 (0.73 to 8.56)	94.7 (86.9 to 98.5)	19.7 (15.6 to 24.2)	13.1	1.11 (0.66 to 1.87)	92.0 (88.7 to 94.5)	20.0 (18.4 to 21.6)
≥33.3	21.1	2.93 (1.49 to 5.77)	75.7 (70.0 to 80.8)	42.0 (37.9 to 46.1)	22.1	2.55 0.75 to 8.71)	82.7 (72.2 to 90.4)	37.3 (32.2 to 42.6)	15.2	1.38 (0.83 to 2.29)	83.9 (79.8 to 87.5)	38.0 (36.1 to 39.9)
≥50.0	27.0	4.52 (2.40 to 8.52)	64.1 (57.9 to 69.9)	57.0 (52.9 to 61.1)	26.8	4.03 (1.26 to 12.93)	70.7 (59.0 to 80.6)	54.6 (49.2 to 60.0)	18.2	2.02 (1.26 to 3.24)	74.8 (70.1 to 79.1)	54.4 (52.4 to 56.4)
≥66.7	30.1	5.35 (2.85 to 10.05)	44.4 (38.3 to 50.7)	76.6 (69.9 to 77.2)	32.4	4.41 (1.37 to 14.16)	52.0 (40.2 to 63.7)	71.7 (66.6 to 76.4)	21.5	3.83 (2.48 to 5.91)	61.4 (56.2 to 66.4)	70.9 (69.1 to 72.7)
≥83.3	35.1	7.93 (4.18 to 15.05)	22.8 (17.8 to 28.4)	89.0 (86.2 to 91.5)	37.5	9.66 (3.15 to 26.65)	33.3 (22.9 to 45.2)	87.3 (45.2 to 90.6)	27.6	6.91 (4.55 to 10.50)	37.3 (32.3 to 42.4)	86.6 (85.2 to 87.9)

Abbreviations: ANU-ADRI, Australian National University–Alzheimer Disease Risk Index; CAIDE, Cardiovascular Risk Factors, Aging, and Dementia; CHS-CS, Cardiovascular Health Study Cognition Study; CogDrisk, Cognitive Health and Dementia Risk Index; HRS-ADAMS, Health and Retirement Study–Aging, Demographics and Memory Study; LIBRA, Lifestyle for Brain Health; MAP, Rush Memory and Aging Project; NA, not applicable; OR, odds ratio.

### Performance of CogDrisk-AD and ANU-ADRI for Estimating AD

The Figure reports the results of the analyses comparing the AUC of CogDrisk-AD and ANU-ADRI for estimating risks of AD in all 3 cohorts. The overall AUC (95% CI) were very similar for estimating AD risks using CogDrisk-AD and ANU-ADRI in all 3 cohorts. The sex-specific AUC (95% CI) was also similar for both male and female participants (see eTable 6 in Supplement 1 for details). The results from the multiple imputed data sets were very similar to that of the available data analysis for both the CogDrisk-AD and ANU-ADRI.

**Figure.**
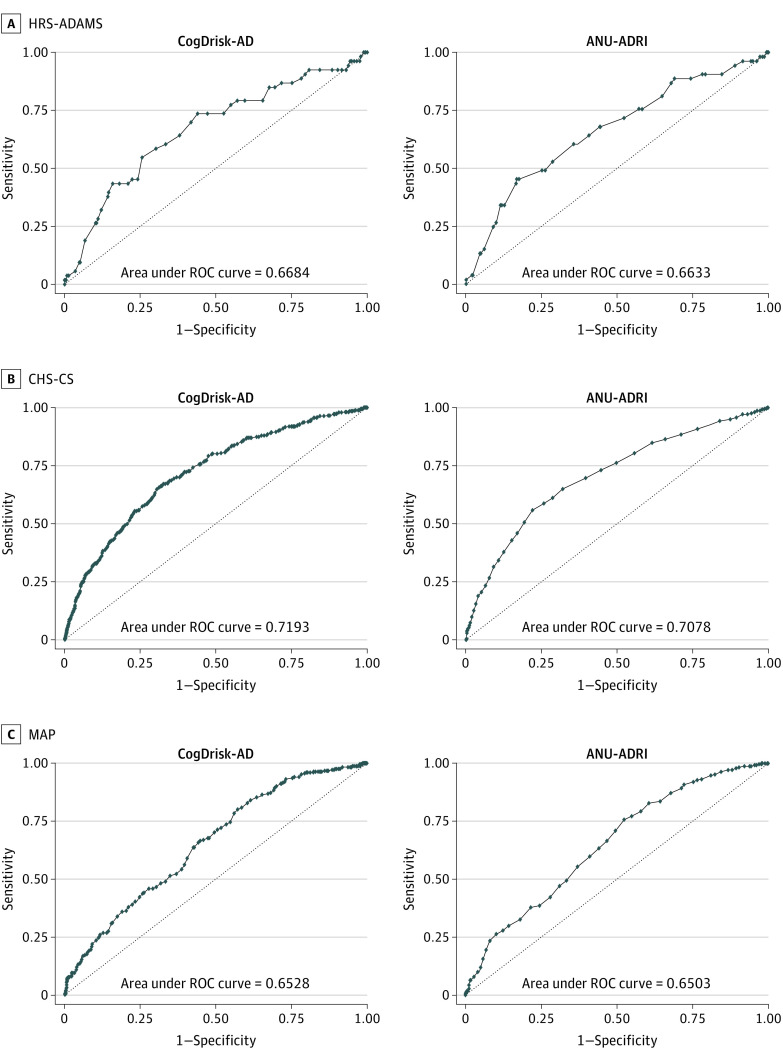
Comparison of the Performance of Cognitive Health and Dementia Risk Index for Alzheimer Disease (CogDrisk-AD) and Australian National University–Alzheimer Disease Risk Index (ANU-ADRI) Risk Scores for Estimating Alzheimer Disease in Multiple Cohorts CHS-CS indicates Cardiovascular Health Study Cognition Study; HRS-ADAMS, Health and Retirement Study–Aging, Demographics and Memory Study; MAP, Rush Memory and Aging Project; ROC, receiver operating characteristic.

## Discussion

In this cohort study, we found that CogDrisk, ANU-ADRI, and modified LIBRA have better assessment of dementia risks compared with CAIDE and LIBRA. Similar findings were also reported in previous studies.^12,13^ LIBRA does not include weights for age, sex, and education in the scoring, hence results in poor performance in estimating dementia risk. The poor performance of CAIDE in our study may also be because it was originally developed as a midlife risk score and none of the study data sets used in this study had sufficient midlife information. In addition, the CAIDE risk score assigned the same risk weight for age among individuals over 53 years of age, which does not take account of the increasing incidence and prevalence of dementia observed in higher age groups,^20^ hence it may be of limited use for assessing dementia risk in older populations. The study data sets had only a few factors associated with increased or reduced risk that could be used for the CAIDE risk score. The low number of risk factors may also have contributed to the poor performance of CAIDE relative to the other risk scores. Similar findings were also reported in previous studies.^6^ CogDrisk covered the widest range of established factors for increased or reduced risk of dementia, followed by ANU-ADRI, LIBRA, and CAIDE. The larger number of factors in CogDrisk may enhance its utility as a tool to inform and monitor risk modification in community settings and in intervention studies. Self-assessment using a tool that includes more modifiable risk factors may also motivate behavioral change because of its greater scope for risk identification and tailoring, and hence may be preferable in risk intervention studies.

We found that each risk score provided very similar AUCs when applied without age, sex, and education, and these AUCs were comparatively low.^21^ This also demonstrated the discriminant power of age, sex, and education in dementia risk assessment. Age and sex, although not modifiable, are the most substantial risk factors for dementia.^13^ As an index of time and risk exposure, age and sex often are proxy indices for latent cumulative risk. All modifiable dementia risk factors increase with age and their exposure is cumulating over time. Similar to the previous studies,^13^ we have found that using a model with only age and sex has similar estimation of dementia risks compared with risk models incorporating demographic, health, and lifestyle risk factors (eTable 7 in Supplement 1). But age itself is a marker of time since birth and has no biological or causative properties, hence age cannot substantively explain anything. Therefore, removing age from risk tools, or using age only in a risk tool, are both counterproductive. This is because the former fails to address the latent cumulative index of age and the latter will not inform any risk reduction strategies. Indeed, other widely used risk scores for heart disease and diabetes, such as Framingham^22^ and AusDrisk,^23^ also include age.

Although there was variability in the factors included in each cohort study, and variation in the prevalence of dementia and/or risk factors across the cohorts, the performance of the models were similar across cohorts. Also, CogDrisk-AD and ANU-ADRI performed similarly in estimating the risk of AD. This is not surprising as both risk scores were developed using very similar risk factors where risk ratios were obtained using very similar methodology.

### Limitations

This study had limitations. One study limitation is the relatively older baseline age of all the cohorts. Hence, some of the midlife risk factor information, such as obesity and high cholesterol, were not used in the analysis. As CAIDE and LIBRA are based on midlife risk factors, further assessment of these risk tools is needed in cohorts that have a long enough follow-up time to cover both midlife risk factors and late life diagnosis of dementia. However, the availability of such studies is limited. Another limitation of this study is that our best risk score considered here only provides poor to acceptable discrimination (AUC between 0.65 and 0.75) (see general guidelines of AUC^21^). However, these results are similar to the AUC for well-established risk estimation models for other diseases, such as stroke, diabetes, and cardiovascular disease.^24,25,26,27,28^ Tools based on these models are widely used in clinical practice; risk tools are used to inform clinicians and patients which risk factors the patient has, and this is used to develop risk reduction advice or treatment. All the risk factors included in these tools are strongly supported by systematic reviews. To our knowledge, there is no current consensus on the level of predictive accuracy for tools used in clinical practice. Dementia risk prediction models that use *APOE* ε4 status or other clinical markers are shown to provide more predictive ability^12,29^ than the risk tools examined in this study. However, inclusion of *APOE* ε4status in CogDrisk and ANU-ADRI did not change the estimative value for dementia in any of the cohorts under investigation (results not shown). Moreover, adding clinical markers or *APOE* ε4 status into the risk tools may increase the accuracy of estimates, but *APOE* ε4 genotyping may not be readily available to be implemented in community settings. The estimating performance of a given risk score depends on the factors included in a model as well as the availability, quality, and completeness of the data. Comparing risk models side by side on the same set of test data provides a clearer picture regarding their relative accuracy.

## Conclusion

This cohort study compared several dementia risk tools alongside each other using multiple well-known dementia cohorts. We found that CogDrisk and CogDrisk-AD provided similar accuracy for estimating future dementia and AD risks as the ANU-ADRI. As CogDrisk and CogDrisk-AD are based on the most up-to-date evidence and include more modifiable risk factors relative to other risk scores in this study, they may have more potential to inform risk modification and/or risk intervention in the community.

## Notes

Supplement 1. **eTable 1.** Description of the Covariates Used in the Various Dementia Risk Assessment Tools **eTable 2.** Descriptive Statistics for the Three Evaluation Cohorts in Terms of Risk and Protective Factors Used in Various Prediction Models **eTable 3.** Comparison of the Performance of Risk Scores for Predicting Dementia in Multiple Cohorts Without Age, Sex, and Education **eTable 4.** Correlation Between Various Risk Scores Across Three Different Cohorts **eTable 5.** Correlation Between Various Risk Scores Excluding Points for Age, Sex, and Education Across Three Different Cohorts **eTable 6.** Comparison on the Performance of CogDrisk-AD and ANU-ADRI Risk Scores for Predicting Alzheimer Disease in Various Cohorts **eTable 7.** Comparison of the Performance of Risk Scores for Predicting Dementia in Multiple Cohorts With Age and Sex Only Model

Supplement 2.
Data Sharing Statement
