# Supplementary material for: CogDrisk, ANU-ADRI, CAIDE, and LIBRA Risk Scores for Estimating Dementia Risk
Source: JAMA Netw Open. 2023 Aug 30;6(8):e2331460. doi: 10.1001/jamanetworkopen.2023.31460 (PMC10469268; doi:10.1001/jamanetworkopen.2023.31460)
Supplement: Supplement 1. — eTable 1. Description of the Covariates Used in the Various Dementia Risk Assessment Tools eTable 2. Descriptive Statistics for the Three Evaluation Cohorts in Terms of Risk and Protective Factors Used in Various Prediction Models eTable 3. Comparison of the Performance of Risk Scores for Predicting Dementia in Multiple Cohorts Without Age, Sex, and Education eTable 4. Correlation Between Various Risk Scores Across Three Different Cohorts eTable 5. Correlation Between Various Risk Scores Excluding Points for Age, Sex, and Education Across Three Different Cohorts eTable 6. Comparison on the Performance of CogDrisk-AD and ANU-ADRI Risk Scores for Predicting Alzheimer Disease in Various Cohorts eTable 7. Comparison of the Performance of Risk Scores for Predicting Dementia in Multiple Cohorts With Age and Sex Only Model [file jamanetwopen-e2331460-s001.pdf]

Supplemental Online Content

Huque MH, Kootar S, Eramudugolla R, et al. CogDrisk, ANU-ADRI, CAIDE, and LIBRA risk scores for estimating dementia risk. *JAMA Netw Open.* 2023;6(8):e2331460. doi:10.1001/jamanetworkopen.2023.31460

eTable 1. Description of the Covariates Used in the Various Dementia Risk Assessment Tools

eTable 2. Descriptive Statistics for the Three Evaluation Cohorts in Terms of Risk and Protective Factors Used in Various Prediction Models

eTable 3. Comparison of the Performance of Risk Scores for Predicting Dementia in Multiple Cohorts Without Age, Sex, and Education

eTable 4. Correlation Between Various Risk Scores Across Three Different Cohorts

eTable 5. Correlation Between Various Risk Scores Excluding Points for Age, Sex, and Education Across Three Different Cohorts

eTable 6. Comparison on the Performance of CogDrisk-AD and ANU-ADRI Risk Scores for Predicting Alzheimer Disease in Various Cohorts

eTable 7. Comparison of the Performance of Risk Scores for Predicting Dementia in Multiple Cohorts With Age and Sex Only Model

This supplemental material has been provided by the authors to give readers additional information about their work.

© 2023 Huque MH et al. *JAMA Network Open.*

eTable 1. Description of the Covariates Used in the Various Dementia Risk Assessment Tools

	**CogD points**	**CogD-AD points**	**ANU-ADRI points**	**LIBRA‡**	**Modified-LIBRA§§**	**CAIDE**
Age for males (years)				NA		
60-64	0	0	0		0	<47=1
65-69	6	5	1		1	47-53=4
70-74	8	8	12		12	>53=5
75-79	13	12	18		18	
80-84	17	17	26		26	
85-89	20	19	33		33	
>90	22	23	38		38	
Age for females (years)				NA		
60-64	0	0	0		0	<47=0
65-69	4	5	5		5	47-53=3
70-74	7	7	14		14	>53=4
75-79	11	13	21		21	
80-84	15	16	29		29	
85-89	19	19	35		35	
>90	23	23	41		41	
Education level, n(%)				NA		
Primary	4	4	6		6	<7; 3
Secondary	2	2	3		3	7-9; 2
Tertiary	0	0	0		0	>=10; 0
Midlife (<=65 years) obesity, n %						
Under weight	2	3	-	-	-	-
Normal	0	0	0	0	0	0
Overweight	1	1	2	1.6	1.6	2
Obese	3	2	5	1.6	1.6	2
Diabetes						NA
Yes	2	2	3	1.3	1.3	
Depression						NA
CESD>16	3	4	2	2.1	2.1	
High Cholesterol (aged <60)						
Yes	3	3	3	1.4	1.4	2
TBI				NA	NA	NA
Yes	2	1	4			
Smoking						NA
Never	0	0	0	0	0	
Former	0	0.2	1	0	0	
Current	1	2	4	1.5	1.5	
Alcohol	NA					
Abstain			0	0	0	
Light-moderate			-3	-1.0	-1.0	

© 2023 Huque MH et al. *JAMA Network Open.*

Social engagement	NA	NA		NA	NA	NA
Lowest			6			
Lowest-med			4			
Med-high			1			
Highest			0			
Loneliness			NA	NA	NA	NA
Yes	2	2				
Physical activity						
Lowest/Physically inactive	0	0	0	1.1[†]	1.1[†]	1
Moderate	-3	-3	-2	0	0	0
Vigorous	-3	-3	-3	0	0	0
Cognitive activity						NA
Lowest	0	0	0	0	0	
Medium	-4	-4	-6	0	0	
Highest	-5	-5	-7	-3.2	-3.2	
Fish intake	NA	NA		NA	NA	NA
<0.25 p p/per week			0			
0.25-2 p p/per week			-3			
2-4 p p/per week			-4			
>4 p/week			-5			
Fish serves per week	-0.25	-0.4	NA			NA
Medi-diet				-1.7	-1.7	
Hypertension (<=65 Years)			NA			
Yes	1	1		1.6	1.6	2
Stroke			NA	NA	NA	NA
Yes	2	2				
Atrial Fibrillation (>65 Years)		NA	NA	NA	NA	NA
Yes	2					
Insomnia		NA	NA	NA	NA	NA
Yes	2					
CHD	NA	NA	NA			NA
Yes				1	1	
Renal dysfunction	NA	NA	NA	1.1	1.1	NA
Pesticides exposure	NA			NA	NA	NA
Yes		2	2			

‡The original LIBRA study followed up mid-late life participants for up to 16 years.
§The original LIBRA plus age, sex and education weights from ANU-ADRI
†LIBRA score for physically inactive people.
NA: Not included in in the tool

© 2023 Huque MH et al. *JAMA Network Open.*

eTable 2. Descriptive Statistics for the Three Evaluation Cohorts in Terms of Risk and Protective Factors Used in Various Prediction Models

	MAP	HRS ADAMS	CHS-CS
Sample size (n)	2184	548	3375
Mean age at baseline in years, (SD)	80·0 (7·6)	79.5 (6.3)	74·8 (4·9)
Age range, years	54-100	70-103	65-97
Follow-up time in years [Median (p25, p75)]†	5 (1, 2)	5 (2, 6)	6.01 (0.19, 7.66)
Males, n (%)	578 (26·5)	260 (47·5)	1381 (40·9)
Age of males (years), n (%)			
<60	3 (0·1)	---	---
60-64	14 (0·6)	---	---
65-69	35 (1·6)	---	130 (3.9)
70-74	61 (2·8)	18 (3.3)	610 (18.1)
75-79	123 (5.6)	82 (15.0)	391 (11.6)
80-84	182 (8·3)	69 (12.6)	181 (5.4)
85-89	113 (5·2)	60 (11.0)	55 (1.6)
>90	47 (2·2)	31 (5.7)	14 (0.4)
Age of females (years), n (%)			
<60	20 (0·9)	---	---
60-64	43 (2·0)	---	---
65-69	127 (5·8)	---	239 (7.1)
70-74	204 (9·3)	18 (3.3)	890 (26.4)
75-79	370 (16·9)	56 (10.2)	521 (15.4)
80-84	432 (19·8)	97 (17·7)	267 (7.9)
85-89	280 (12·8)	64 (11·7)	69 (2.0)
>90	130 (6·0)	53 (10·7)	8 (0·2)
Education level, n (%)			
Primary	70 (3·2)	166 (30.3)	368 (10·9)
Secondary	87 (4·0)	223 (40·7)	442 (13·1)
Tertiary	2027 (92·8)	159 (29·0)	2560 (75·9)
Missing	.	.	5 (0·2)
Midlife (<=65 years) Obesity, n (%)		NA	NA
Under weight	-		
Normal	18 (0·8)		
Overweight	26 (1·2)		
Obese	33 (1·5)		
Missing	2107 (96·4)		
Diabetes, n (%)			
Yes	274 (12·6)	124 (22.6)	508 (15·1)
Missing	117 (5·4)	4 (0.7)	76 (2·3)
Depression, n (%)			
Yes	218 (10·0)	6 (1·1)	612 (18·1)
Missing	119 (5·5)	7 (1.3)	---
Midlife High Cholesterol >6.5mmol/litre	NA	NA	NA
TBI, n (%)			NA
Yes	131 (6·0)	27 (4.9)	
Missing	275 (12·6)	50 (9·1)	
Smoking, n (%)			
Never smoked	1251 (57·6)	251 (45.8)	1507 (44·7)
Former smoker	862 (39·7)	232 (42.3)	1555 (46·1)
Current smoker	58 (2·7)	60 (11·0)	312 (9·2)
Missing	13 (0·6)	5 (0.9)	----
Alcohol consumption, n (%)			
Abstain	1152 (52.8)	175 (31.9)	1592 (47.2)

© 2023 Huque MH et al. *JAMA Network Open.*

Light-moderate	900 (41.2)	285 (52.0)	1534 (45.5)
missing	16 (0.7)	4 (0.7)	10 (0.3)
Social engagement, n (%)		NA	NA
Lowest	620 (28.4)		
Lowest-med	624 (28.5)		
Med-high	466 (21.3)		
Highest	449 (20.6)		
Missing	26 (1.2)		
Loneliness, n (%)		NA	
Yes	177 (8·1)		266 (7·9)
Missing	317 (14·5)		12 (0·4)
Physical activity, n (%)		NA	
Moderate/vigorous	1370 (62·7)		2130 (63·1)
Missing	0 (0·0)		8 (0·2)
Cognitive stimulating activity, n (%)			N/A
Low	826 (37·8)	349 (63.7)	
Moderate	1058 (48·4)	110 (20.1)	
High	182 (8·3)	19 (3.5)	
Missing	118 (5·4)	70 (12.8)	
Fish intake			
<0.25 p p/per week	0 (0.0)	NA	590 (17.5)
0.25-2 p p/per week	123 (5.6)		1216 (36.0)
2-4 p p/per week	503 (23.0)		900 (80.2)
>4 p/week	437 (20.0)		669 (19.8)
Missing	1121 (51·3)		0 (0.0)
Fish serves/week, median (ranges)	4 (2-11)	NA	1·5 (0-17)
Missing	1121 (51·3)		---
Hypertension (<=65 Years)		NA	NA
Yes	33 (0.02)		
Missing	2180 (96.3)		
Stroke, n (%)			
Yes	169 (7·7)	76 (13.9)	152 (4·5)
Missing	285 (13·1)	7 (1·3)	
Atrial fibrillation, n (%)	NA		
Yes		12 (2.2)	97 (2·9)
Missing		335 (61·1)	
Insomnia, n (%)	NA		
Yes		2 (0·4)	991 (29·4)
Missing		3 (0·6)	58 (1·7)
Coronary heart disease, n (%)			
Yes	195 (8.9)	86 (15.7)	539 (16.0)
Missing	3 (0.1)	5 (0.9)	0 (0.0)
Renal dysfunction, n (%)	NA	NA	
Yes			37 (1.1)
Missing			128 (3.8)
Pesticides exposure, n(%)	NA	NA	NA
Dementia, n (%)			
Yes	589 (27·0)	106 (19.3)	480 (14·2)
Alzheimer's disease			
Yes	571 (26.1)	77 14.1)	396 (11.7)
Missing	0 (0.0)	0 (0.0)	599 (17.8)

Abbreviations:
TBI: Traumatic brain injury
BMI: Body mass index
p25: 25th Percentile
p75: 75th Percentile.
Note: Percentage was calculated based on total sample size.

© 2023 Huque MH et al. *JAMA Network Open.*

eTable 3. Comparison of the Performance of Risk Scores for Predicting Dementia in Multiple Cohorts Without Age, Sex, and Education

			CogDrisk	ANU-ADRI	CAIDE	LIBRA
MAP		Variables	(11)[‡] Obesity[†], Diabetes, Depression, TBI, Smoking, Loneliness, Physical activity, Cognitive activity, Fish intake, stroke and hypertension[†]	(10) Obesity[†], Diabetes, Depression, TBI, Smoking, Social network, Physical activity, Cognitive activity, Fish intake, and alcohol intake	(4) Obesity[†], TBI, Physical activity and hypertension[†]	(9) Obesity[†], Diabetes, Depression, Smoking, Physical activity, Cognitive activity, Alcohol intake, hypertension[†] and coronary heart disease.
	Available data analysis	n	N=848, M=210, F=638	N=848, M=210, F=638	N=848, M=210, F=638	N=848, M=210, F=638
		AUC Male	0.56 (0.48, 0.65)	0.51 (0.42, 0.60)	0.55 (0.48, 0.62)	0.57 (0.49, 0.66)
		AUC Female	0.53 (0.48, 0.58)	0.55 (0.50, 0.60)	0.49 (0.44, 0.53)	0.50 (0.46, 0.55)
		AUC Overall	0.54 (0.49, 0.58)	0.54 (0.50, 0.58)	0.50 (0.47, 0.54)	0.52 (0.48, 0.56)
	MI analysis	n	N=2172, M=574, F=1598	N=2172, M=574, F=1598	N=2172, M=574, F=1598	N=2172, M=574, F=1598
		AUC Male	0.57 (0.51, 0.62)	0.53 (0.47, 0.58)	0.53 (0.48, 0.58)	0.56 (0.51, 0.61)
		AUC Female	0.53 (0.50, 0.57)	0.53 (0.50, 0.56)	0.51 (0.48, 0.54)	0.49 (0.46, 0.52)
		AUC Overall	0.54 (0.52, 0.57)	0.53 (0.50, 0.56)	0.50 (0.47, 52)	0.53 (0.50, 0.55)
HRS-ADAMS		Variables	(7) Diabetes, Depression, TBI, Smoking, Cognitive activity, and stroke	(7) Diabetes, Depression, TBI, Smoking, Cognitive activity, and alcohol intake	(2) TBI, and hypertension	(7) Diabetes, Depression, Smoking, Cognitive activity, Alcohol intake, Hypertension and coronary heart disease.
	Available data analysis	n	N=421, M=200, F=221	N=421, M=200, F=221	N=421, M=200, F=221	N=421, M=200, F=221
		AUC Male	0.58 (0.44, 0.71)	0.50 (0.38, 0.62)	0.58 (0.47, 0.69)	0.50 (0.39, 0.61)
		AUC Female	0.57 (0.47, 0.66)	0.55 (0.46, 0.64)	0.48 (0.40, 0.55)	0.57 (0.48, 0.66)
		AUC Overall	0.54 (0.47, 0.63)	0.51 (0.44, 0.58)	0.51 (0.44, 0.57)	0.52 (0.45, 0.59)
	MI analysis	n	N=547, M=260, F=287	N=547, M=260, F=287	N=547, M=260, F=287	N=547, M=260, F=287
		AUC Male	0.54 (0.45, 0.64)	0.50 (0.40 0.59)	0.52 (0.43, 0.61)	0.56 (0.47, 0.66)
		AUC Female	0.53 (0.45, 0.61)	0.52 (0.44, 0.61)	0.54 (0.47, 0.61)	0.52 (0.42, 0.60)
		AUC Overall	0.52 (0.46, 0.58)	0.53 (0.47, 0.59)	0.53 (0.48, 0.58)	0.53 (0.47, 0.58)
CHS-CS		Variables	(9) Diabetes, Depression, Smoking, physical activity, loneliness, Insomnia, stroke, Atrial Fibrillation and fish intake	(6) Diabetes, Depression, Smoking, Alcohol intake, physical activity, and fish intake	(1) Physical activity	(7) Diabetes, Depression, Smoking, Alcohol intake, physical activity, coronary heart disease and renal dysfunction
	Available data analysis	n	N=3100, M=1263, F=1837	N=3100, M=1263, F=1837	N=3100, M=1263, F=1837	N=3100, M=1263, F=1837
		AUC Male	0.58 (0.53, 0.62)	0.49 (0.44, 0.53)	0.53 (0.49, 0.56)	0.51 (0.46, 0.56)
		AUC Female	0.56 (0.52, 0.59)	0.49 (0.45, 0.52)	0.54 (0.51, 0.57)	0.51 (0.47, 0.55)

© 2023 Huque MH et al. *JAMA Network Open.*

		AUC Overall	0.56 (0.53, 0.59)	0.49 (0.46, 0.52)	0.53 (0.51, 0.56)	0.51 (0.48, 0.54)
	MI analysis	n	N=3370, M=1381, F=1989	N=3370, M=1381, F=1989	N=3370, M=1381, F=1989	N=3370, M=1381, F=1989
		AUC Male	0.59 (0.55, 0.64)	0.50 (0.46, 0.55)	0.54 (0.50, 0.57)	0.56 (0.51, 0.60)
		AUC Female	0.55 (0.51, 0.59)	0.54 (0.50, 0.57)	0.53 (0.50, 0.57)	0.53 (0.49, 0.56)
		AUC Overall	0.57 (0.54, 0.60)	0.52 (0.50, 0.55)	0.54 (0.51, 0.56)	0.54 (0.51, 0.57)

Note: (#)‡: represents the number of number of risk/protective factors

© 2023 Huque MH et al. *JAMA Network Open.*

eTable 4. Correlation Between Various Risk Scores Across Three Different Cohorts

			Correlation-coefficients				
		Median (smallest, largest)	CogDrisk	ANU-ADRI	CAIDE	LIBRA	Modified-LIBRA
MAP	CogDrisk	10.25 (-8.5, 30.25)	1.00				
	ANU-ADRI	20 (-15, 50)	0.89	1.00			
	CAIDE	5 (4, 9)	0.12	-0.03	1.00		
	LIBRA	0 (-4.3, 7.1)	0.29	0.18	0.35	1.00	
	Modified-LIBRA	26.9 (-1.7, 48.1)	0.86	0.92	-0.13	0.09	1.00
HRS-ADMS	CogDrisk	16 (2, 32)	1.00				
	ANU-ADRI	25 (4, 51)	0.94	1.00			
	CAIDE	6 (4, 12)	0.31	0.21	1.00		
	LIBRA	1.5 (-3.2, 5.4)	0.21	0.20	0.35	1.00	
	Modified-LIBRA	26.6 (10.3, 49.6)	0.85	0.94	0.17	0.12	1.00
CHS-CS	CogDrisk	9.45 (-1.25, 29.25)	1.00				
	ANU-ADRI	14 (-9, 44)	0.83	1.00			
	CAIDE	5 (4, 9)	0.47	0.25	1.00		
	LIBRA	1.45 (-1, 7)	0.42	0.35	0.22	1.00	
	Modified-LIBRA	18.1 (0, 48.1)	0.86	0.96	0.24	0.22	1.00

eTable 5. Correlation Between Various Risk Scores Excluding Points for Age, Sex, and Education Across Three Different Cohorts

		CogDrisk	ANU-ADRI	CAIDE	LIBRA
MAP	CogDrisk	1.00			
	ANU-ADRI	0.68	1.00		
	CAIDE	0.48	0.35	1.00	
	LIBRA	0.63	0.52	0.36	1.00
HRS-ADMS	CogDrisk	1.00			
	ANU-ADRI	0.84	1.00		
	CAIDE	-0.17	0.03	1.00	
	LIBRA	0.29	0.55	0.43	1.00
CHS-CS	CogDrisk	1.00			
	ANU-ADRI	0.33	1.00		
	CAIDE	0.60	0.38	1.00	
	LIBRA	0.62	0.76	0.37	1.00

© 2023 Huque MH et al. *JAMA Network Open.*

eTable 6. Comparison on the Performance of CogDrisk-AD and ANU-ADRI Risk Scores for Predicting Alzheimer Disease in Various Cohorts

			CogDrisk-AD	ANU-ADRI
MAP		Variables	(14) Age, Gender, Education, obesity†, Diabetes, Depression, TBI, Smoking, Loneliness, Physical activity, Cognitive activity, Fish intake, stroke and hypertension†	(13) Age, Gender, Education, obesity†, Diabetes, Depression, TBI, Smoking, Social network, Physical activity, Cognitive activity, Fish intake, and alcohol intake
	Available data analysis	n	N=848, M=210, F=638	N=848, M=210, F=638
		AUC Male	0.66 (0.59, 0.74)	0.63 (0.55, 0.71)
		AUC Female	0.65 (0.60, 0.70)	0.66 (0.61, 0.70)
		AUC Overall	0.65 (0.61, 0.69)	0.65 (0.61, 0.69)
	MI analysis	n	N=2172, M=574, F=1598	N=2172, M=574, F=1598
		AUC Male	0.67 (0.63, 0.72)	0.65 (0.60, 0.69)
		AUC Female	0.64 (0.61, 0.67)	0.65 (0.62, 0.68)
		AUC Overall	0.65 (0.62, 0.68)	0.64 (0.62, 0.67)
HRS-ADAMS		Variables	(9) Age, Gender, Education, Diabetes, Depression, TBI, Smoking, Cognitive activity, and stroke	(9) Age, Gender, Education, Diabetes, Depression, TBI, Smoking, Cognitive activity, and alcohol intake
	Available data analysis	n	N=424, M=201, F=223	N=424, M=201, F=223
		AUC Male	0.70 (0.55, 0.84)	0.67 (0.52, 0.82)
		AUC Female	0.66 (0.56, 0.76)	0.65 (0.55, 0.75)
		AUC Overall	0.67 (0.59, 0.75)	0.66 (0.58, 0.75)
	MI analysis	n	N=547, M=260, F=287	N=547, M=260, F=287
		AUC Male	0.67 (0.57, 0.77)	0.67 (0.56 0.78)
		AUC Female	0.66 (0.57, 0.75)	0.61 (0.52, 0.70)
		AUC Overall	0.64 (0.58, 0.71)	0.64 (0.58, 0.71)
CHS		Variables	(10) Age, Gender, Education, Diabetes, Depression, Smoking, physical activity, loneliness, stroke, and fish intake	(9) Age, Gender, Education, Diabetes, Depression, Smoking, Alcohol intake, physical activity, and fish intake
	Available data analysis	n	N=2692, M=1100, F=1592	N=1692, M=1100, F=1592
		AUC Male	0.72 (0.68, 0.77)	0.71 (0.67, 0.76)
		AUC Female	0.72 (0.69, 0.76)	0.70 (0.67, 0.74)
		AUC Overall	0.72 (0.69, 0.75)	0.71 (0.68, 0.74)
	MI analysis	n	N=2771, M=1132, F=1639	N=2771, M=1381, F=1989
		AUC Male	0.73 (0.69, 0.78)	0.72 (0.67, 0.76)
		AUC Female	0.73 (0.70, 0.77)	0.71 (0.68, 0.75)
		AUC Overall	0.73 (0.70, 0.76)	0.72 (0.69, 0.75)

© 2023 Huque MH et al. *JAMA Network Open.*

eTable 7. Comparison of the Performance of Risk Scores for Predicting Dementia in Multiple Cohorts With Age and Sex Only Model

		CogDrisk	ANU-ADRI	CAIDE
MAP	n	N=2184, M=578, F=1606	N=2184, M=578, F=1606	
	AUC Male	0.64 (0.60, 0.69)	0.64 (0.60, 0.69)	
	AUC Female	0.65 (0.62, 0.68)	0.65 (0.62, 0.68)	
	AUC Overall	0.65 (0.63, 0.68)	0.65 (0.62, 0.67)	0.52 (0.50, 0.54)
HRS-ADAMS	n	N=548, M=260, F=288	N=548, M=260, F=288	N=548, M=260, F=288
	AUC Male	0.65 (0.56, 0.75)	0.66 (0.57, 0.74)	
	AUC Female	0.59 (0.51, 0.67)	0.58 (0.50, 0.66)	
	AUC Overall	0.61 (0.55, 0.67)	0.63 (0.57, 0.68)	0.45 (0.39, 0.50)
CHS-CS	n	N=3375, M=1381, F=1994	N=3375, M=1381, F=1994	N=3375, M=1381, F=1994
	AUC Male	0.67 (0.62, 0.71)	0.67 (0.62, 0.71)	
	AUC Female	0.71 (0.67, 0.74)	0.71 (0.67, 0.74)	
	AUC Overall	0.69 (0.66, 0.71)	0.69 (0.66, 0.72)	0.50 (0.47, 0.52)

© 2023 Huque MH et al. *JAMA Network Open.*
