# Supplementary material for: CogDrisk, ANU-ADRI, CAIDE, and LIBRA Risk Scores for Estimating Dementia Risk
Source: JAMA Netw Open. 2023 Aug 30;6(8):e2331460. doi: 10.1001/jamanetworkopen.2023.31460 (PMC10469268; doi:10.1001/jamanetworkopen.2023.31460)
Supplement: Supplement 2. — Data Sharing Statement [file jamanetwopen-e2331460-s002.pdf]

Data Sharing Statement

Huque. CogDrisk, ANU-ADRI, CAIDE, and LIBRA Risk Scores for Estimating Dementia Risk. *JAMA Netw Open*. Published August 30, 2023. doi:10.1001/jamanetworkopen.2023.31460

Data
Data available: No

Additional Information
Explanation for why data not available: We have used secondary data analysis. Data may be available from data custodians. MAP data can be requested at www.radc.rush.edu
